# Mutations and thrombosis in essential thrombocythemia

**DOI:** 10.1038/s41408-021-00470-y

**Published:** 2021-04-27

**Authors:** Paola Guglielmelli, Naseema Gangat, Giacomo Coltro, Terra L. Lasho, Giuseppe Gaetano Loscocco, Christy M. Finke, Erika Morsia, Benedetta Sordi, Natasha Szuber, Curtis A. Hanson, Animesh Pardanani, Alessandro M. Vannucchi, Ayalew Tefferi

**Affiliations:** 1 Department of Experimental and Clinical Medicine, CRIMM, Center of Research and Innovation of Myeloproliferative Neoplasms, Azienda Ospedaliera Universitaria Careggi, University of Florence, Florence, Italy; 2 Divisions of Hematology, Mayo Clinic, Rochester, MN USA; 3 Divisions of Hematopathology, Departments of Internal Medicine and Laboratory Medicine, Mayo Clinic, Rochester, MN USA

**Keywords:** Cancer genetics, Myeloproliferative disease

Dear Editor,

Essential thrombocythemia (ET) constitutes one of the three *JAK2*/*MPL*/*CALR*-mutated myeloproliferative neoplasms (MPNs), which also include polycythemia vera (PV) and primary myelofibrosis (PMF)^1^. ET is defined by clonal thrombocytosis (platelet count ≥450 × 10^9^/L) and characteristic bone marrow megakaryocyte morphology; the clinical phenotype in ET might include leukocytosis, splenomegaly, microvascular symptoms, and thrombohemorrhagic complications^1^. Most patients with ET enjoy a near-normal life expectancy while disease progression into fibrotic or leukemic transformation is relatively infrequent (<1% in the first 10 years of disease)^2^. Collaborative studies between the Mayo Clinic, Rochester, MN, USA and University of Florence, Florence, Italy have recently established an integrated clinical and genetic survival risk model for ET^3^. According to this new mutation-enhanced international prognostic model for ET (MIPSS-ET), independent risk factors for overall survival included *SF3B1*/*SRSF2* mutations (occurring in ~ 10% of patients), age >60 years, male sex, and leukocytosis (≥11 × 10^9^/L). To date, drug therapy has not been shown to influence survival in ET and is instead directed at decreasing the risk of thrombosis. In the latter regard, four risk categories are recognized: very low risk (age ≤ 60 years, no thrombosis history, *JAK2* wild type), low risk (age ≤ 60 years, no thrombosis history, *JAK2* mutated), intermediate risk (age > 60 years, no thrombosis history, *JAK2* wild type) and high risk (thrombosis history or age >60 years with *JAK2* mutation)^1^. In the current two-center study involving 502 patients (seen between 1967 through 2017) with World Health Organization (WHO)-defined ET, we sought to further clarify the influence of driver and other mutations on both arterial and venous thrombosis, in the context of other established risk factors.

Study patients were recruited from the Mayo Clinic, Rochester, MN, USA and the University of Florence, Florence, Italy, based on the availability of next-generation sequencing (NGS)-derived mutation information (Table 1). Diagnosis of ET was based on the 2016 WHO criteria^4^. Methods for mutation screening have previously been published; a detailed account of all mutations investigated including their variant allele frequency has previously been published^3,5^. Conventional statistics was employed to examine the significance of associations, with separate analysis of arterial vs venous thrombosis, occurring at any time before or after formal diagnosis of ET. For the purposes of the current study, only the first events were considered.

**Table 1:** Clinical and laboratory characteristics of 270 Mayo Clinic patients and 232 University of Florence patients with essential thrombocythemia (ET) (total *n* = 502).

Variables	Mayo Clinic (*n* = 270)	Florence (*n* = 232)
Age in years; median (range)	57 (18–92)	54 (13–85)
Males; *n* (%)	108 (40)	96 (41)
Hemoglobin, g/dl; median (range) “N” evaluable = 382 (92%)	13.7 (6.9–17.9)^a^	14.1 (12–17.0)^a^
Platelets, ×10^9^/L; median (range) “N” evaluable = 407 (98%)	844 (451–3330)	739 (451–2000)
Platelets > 1000 × 10^9^/l; *n* (%) “N” evaluable = 407 (98%)	82 (31)	33 (16)
Leukocytes, ×10^9^/L; median (range) “N” evaluable = 399 (96%)	8.7 (2.7–70.7)	8.5 (3.8–26)
Leukocytes ≥ 11 × 10^9^/l; *n* (%) “N” evaluable = 399 (96%)	64 (25)	36 (19)
Palpable splenomegaly “N” evaluable = 412 (99%)	48 (18)	45 (21)
Karyotype “N” evaluable = 345 (83%)		
Abnormal; *n* (%)	20 (9)	15 (10)
Fibrotic progression; *n* (%)	44 (16)	76 (33)^b^
Leukemic transformations; *n* (%)	12 (4)	15 (6.5)
Follow up in years; median (range)	9.9 (0–34.6)	12.9 (1–36.3)
Deaths; *n* (%)	104 (39)	87 (38)
**Mutations**^c^		
*JAK2* mutated; *n* (%)	146 (54)	129 (56)
CALR mutated; *n* (%)	79 (29)	59 (25)
*TET2* mutated; *n* (%)	25 (9)	28 (11)
*ASXL1* mutated; *n* (%)	18 (7)	47 (20)^b^
*DNMT3A* mutated; *n* (%)	19 (7)	14 (7)
*SF3B1* mutated; *n* (%)	14 (5)	12 (5)
*SH2B3* mutated; *n* (%)	3 (1)	6 (3)
*SRSF2* mutated*; n* (%)	6 (2)	6 (3)
*MPL* mutated; *n* (%)	11 (4)	17 (7)
*KIT* mutated; *n* (%)	5 (2)	2 (1)
*IDH2* mutated; *n* (%)	4 (1)	2 (1)
*TP53* mutated; *n* (%)	5 (2)	9 (4)
*U2AF1* mutated; *n* (%)	3 (1)	6 (2.5)
*RUNX1* mutated; *n* (%)	4 (1)	5 (2)
*EZH2* mutated; *n* (%)	5 (2)	9 (4)
*CBL* mutated; *n* (%)	3 (1)	3 (2)

^a^ET patients who had low hemoglobin, presented with concomitant bleeding disorders, iron deficiency anemia, chronic renal insufficiency, or other rare disorders such as sickle cell anemia and Osler-Weber-Rendu disease.

^b^The difference in the frequency of ASXL1 mutation and fibrotic transformation in the two patient cohorts is explained by the intentional enrichment of the Florence cohort with patients who had transformed to myelofibrosis for the purposes of a prior project examining the predictive value of mutations for post-ET fibrotic transformation.

^c^Included mutations with frequency of at least 1%. Also, the denominator for the percentages in parenthesis is the number of evaluable cases.

A total of 502 patients (median age 55 years; 41% above age 60; females 59%) who were molecularly annotated for driver mutations as well as other mutations derived from NGS data were followed for a median of 10 years (0.1–34.7). The Mayo/Florence cohorts included 270/232 patients (median age 57/54 years, 60%/59% females) with median follow-up of 9.9/12.9 years (Table 1). Overall driver mutation distribution was 55% *JAK2*, 27% *CALR*, 5% *MPL*, and 13% triple-negative (TN); among 132 *CALR*-mutated cases, 78 (59%) harbored type-1/like and 54 (41%) type-2/like *CALR* mutations^6^. Most frequent mutations, other than *JAK2*/*CALR*/*MPL*, were *ASXL1* (7–20%), *TET2* (9–11%), *DNMT3A* (7%), *SF3B1* (5%), *SRSF2* (2–3%), *EZH2* (2–4%), *TP53* (2–4%), *RUNX1* (1–2%), *CBL* (1–2%), *IDH2* (1%) and *U2AF1* (1%). Leukocytosis (≥11 × 10^9^/L) was documented in 22% of patients, extreme thrombocytosis (≥1000 × 10^9^/L) in 27% and abnormal karyotype in 9%. Patients were managed according to conventional strategies including cytoreductive drugs for high-risk disease and antiplatelet therapy for low-risk disease.

History of thrombosis (arterial or venous) before or after diagnosis was documented in 152 (30%) patients and included arterial events in 96 (19%) and venous in 82 (16%). The number of arterial/venous vascular events before and after the time of formal diagnosis were 50 (10%)/3 (1%) and 87 (17%)/76 (15%). In univariate logistic regression analysis the incidence of all thrombotic events (i.e. arterial or venous) occurring before or after diagnosis was significantly associated with driver mutation profile (*p* < 0.01; *JAK2* 36%, *CALR* 20%, *MPL* 26%, and TN 32%) and the absence of *ASXL1* (*p* = 0.04) or *IDH* mutations (*p* = 0.01). A similar analysis restricted to arterial events occurring before or after diagnosis revealed a near-significant association for driver mutation profile (*p* = 0.2; *JAK2* 22%, *CALR* 14%, *MPL* 22%, and TN 19%) and significant associations for the absence of *ASXL1* (*p* = 0.02) or *RUNX1* (*p* = 0.05) mutations; a similar analysis for venous events marked driver mutation profile (*p* = 0.03; *JAK2* 21%, *CALR* 10%, *MPL* 15% and TN 13%) and absence of *SRSF2* (*p* = 0.03) or *EZH2* (*p* = 0.02) mutations as being significant. In a subsequent multivariable logistic regression analysis that included age >60 years and leukocytosis (≥11 × 10^9^/L), significance was confirmed for *JAK2* vs *CALR* (*p* < 0.01), absence of *IDH* (0.01) or *ASXL1* (*p* = 0.06) mutations, for all thrombosis; *JAK2* vs *CALR* (*p* < 0.01) and absence of *SRSF2* (*p* = 0.02) or *EZH2* (*p* = 0.03) mutations, for venous thrombosis; and absence of *ASXL1* (*p* = 0.02) or *RUNX1* (*p* = 0.05) mutations for arterial events; of note, in all instances, significant associations were not apparent for either leukocytosis or age >60 years.

Next, in order to mitigate the confounding effect of treatment, we queried for associations with thrombotic events occurring at or before the time of diagnosis; during such analysis, extreme thrombocytosis (≥1000 × 10^9^/L) was also included as variable of interest, based on previous reports of its association with lower risk of arterial thrombosis^7^ as well as *CALR* mutations^8^. In univariate analysis, the following were found to be significantly or near-significantly associated with an increased risk of arterial events: absence of extreme thrombocytosis (*p* = 0.03; 12% vs 5%), age >60 years (*p* = 0.04), absence of *ASXL1* (*p* = 0.09), *EZH2* (*p* = 0.08), *RUNX1* (*p* = 0.17) mutations, wild-type *ASXL1/RUNX1/EZH2* genotype (*p* = 0.03; 9% vs 1%), and driver mutation profile (*p* = 0.09; *JAK2* 11%, *CALR* 5%, *MPL* 19%, and TN 13%); leukocytosis was not significant (*p* = 0.8); multivariable analysis confirmed significance of wild-type *ASXL1*/*RUNX1*/*EZH2* genotype (*p* = 0.03), age >60 years (*p* = 0.05) and absence of extreme thrombocytosis (*p* = 0.05), while driver mutation profile was relegated to borderline significance (*p* = 0.09). A similar analysis for venous events did not reveal any significant association. Finally, for arterial vascular events occurring after diagnosis (i.e. in the context of ongoing anti-thrombotic therapy), logistic regression analysis identified *JAK2* vs *CALR* (*p* < 0.01) and wild-type *ASXL1*/*RUNX1*/*EZH2* genotype (*p* = 0.07) as being significant or near-significant. Cox progression analysis for arterial thrombosis-free survival was feasible in the Mayo Clinic cohort and confirmed the significant unfavorable effect of wild-type *ASXL1*/*RUNX1*/*EZH2* genotype (*p* = 0.02), and also identified a previous arterial event as an independent risk factor (*p* = 0.04).

**Fig. 1:**
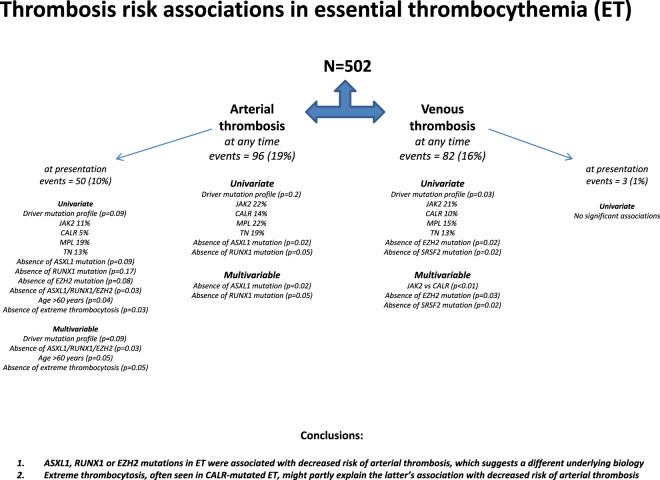
Thrombosis risk associations in essential thrombocythemia.

Figure 1 summarizes our findings in the current study. Our main observations included (i) salutary effect of *ASXL1*/*RUNX1*/*EZH2* mutations on the risk of arterial thrombosis in ET and (ii) prognostic interaction between extreme thrombocytosis and *CALR* mutation in influencing the incidence of arterial events at the time of diagnosis. The favorable influence of harboring *ASXL1*/*RUNX1*/*EZH2* mutations on arterial thrombosis was evident in the context of arterial events occurring both before and after diagnosis. To that effect, it is reasonable to consider the possibility that the infrequent occurrence of high-risk mutations might be a marker for a biologically different disease phenotype, such as occult prefibrotic myelofibrosis^9^. In a previous report in ET, overall survival was adversely affected by spliceosome (*SF3B1*, *SRSF2*) and leukemia-free survival by *TP53* mutations^3^. In a more recent report of young patients with ET, extreme thrombocytosis was independently associated with shortened overall and leukemia-free survival whereas there was no such influence from driver mutations including *JAK2* or *CALR*^10^.

Extreme thrombocytosis in ET has previously been associated with both lower risk of arterial thrombosis^7^ and *CALR* mutations^8^. *CALR* mutations in ET have also been associated with decreased risk of thrombosis, which in the past has been attributed to the younger age distribution of affected cases^11,12^. The current study confirms the prothrombotic influence of *JAK2*, as opposed to *CALR* mutation^13^, and suggests that extreme thrombocytosis might also play a part in contributing to the observed decreased risk of arterial thrombosis in *CALR*-mutated ET. Additional studies are required to confirm these observations, assess their clinical impact, and provide additional insight into the underlying biological mechanisms, which are likely to include the long-recognized, platelet count-dependent, development of acquired von Willebrand syndrome^14^.
